# The effects of age at correction of aortic coarctation and recurrent obstruction on adolescent patients: MRI evaluation of wall shear stress and pulse wave velocity

**DOI:** 10.1186/s41747-019-0102-9

**Published:** 2019-06-20

**Authors:** Joe F. Juffermans, Ineke Nederend, Pieter J. van den Boogaard, Arend D. J. ten Harkel, Mark G. Hazekamp, Hildo J. Lamb, Arno A. W. Roest, Jos J. M. Westenberg

**Affiliations:** 1 Department of Radiology, Leiden University Medical Center, Albinusdreef 2, 2333 ZA Leiden, the Netherlands; 2 Department of Pediatric Cardiology, Leiden University Medical Center, Albinusdreef 2, 2333 ZA Leiden, the Netherlands; 3 Department of Cardiothoracic Surgery, Leiden University Medical Center, Albinusdreef 2, 2333 ZA Leiden, the Netherlands

**Keywords:** Adolescent, Aortic coarctation, Bicuspid aortic valve, Magnetic resonance imaging, Pulse wave analysis

## Abstract

**Background:**

Coarctation patients before curative reconstruction are exposed to abnormal flow patterns which potentially could cause wall deterioration. This study evaluated the effect of age at correction on the pulse wave velocity (PWV) and peak wall shear stress (WSS) in adolescent patients with corrected coarctation. Effects of valve morphology and presence of reobstruction were also evaluated.

**Methods:**

Twenty-one patients aged 13.7 ± 2.6 years (mean ± standard deviation) were included (bicuspid aortic valve, *n* = 14; reobstruction, *n* = 9). Mean age at correction was 1.0 ± 1.8 years. PWV was determined from two high-temporal through-plane phase-contrast magnetic resonance imaging (MRI) acquisitions, for two segments: ascending aorta plus aortic arch and descending aorta. WSS was determined from four-dimensional flow MRI. Peak WSS over five systolic phases was determined for ascending aorta, aortic arch, and descending aorta.

**Results:**

Patients with tricuspid aortic valve showed a significant correlation between the age at correction and descending aorta PWV (*r*_**s**_ = 0.80, *p* = 0.010). Significant differences were found between patients without and with reobstruction for peak WSS in the aortic arch (3.9 ± 1.3 Pa *versus* 6.5 ± 2.2 Pa, respectively; *p* = 0.003) and descending aorta (5.0 ± 1.3 Pa *versus* 6.7 ± 1.1 Pa, respectively; *p* = 0.005).

**Conclusions:**

A prolonged period of abnormal haemodynamic exposure may result in increased aortic wall stiffening. The increased peak WSS as results of a reobstruction possibly promotes different disease progression, which endorse longitudinal follow-up examination of corrected coarctation patients.

## Key points

- Coarctation correction age correlates with descending aorta pulse wave velocity in patients with tricuspid aortic valve.
- Recurrent obstruction induces higher peak wall shear stress in the aortic arch.
- Recurrent obstruction induces higher peak wall shear stress in the descending aorta.

## Background

Aortic coarctation (CoA) is a congenital obstruction of aorta [1–3], typically located just distally from the aortic arch [2–7]. With a prevalence of approximately 3 to 4 per 10,000 live births [7–9], CoA accounts for 5 to 8% of all congenital heart defects [1–3, 6]. The most commonly associated abnormality is a bicuspid aortic valve (BAV), with a prevalence rate between 60 and 85% in patients with CoA [3–6, 8]. After curative reconstruction, patients are at risk to develop late hypertension and residual or recurrent obstruction [3, 10], the latter with a prevalence up to 30% [4].

Before reconstruction, the local aortic narrowing results in an increased afterload of the heart and a pressure gradient over the obstruction with associated formation of abnormal aorta flow patterns [1]**.** The viscous friction of the blood (*i.e*., the haemodynamic load) on the vessel wall regulates the endothelium lining properties, which by high wall shear stress (WSS) promotes vascular dilatation and remodelling [11]. Additionally, more collagen tissue and less smooth muscle fibres are observed within the aortic wall proximally towards the lesion compared to distally [12]. It is unclear whether the abnormal haemodynamic situation prior to the correction already causes aortic wall deterioration [13]. Knowledge of this causality is important in order to identify the ideal time for intervention [7]. Paradoxically, an older age of curative CoA reconstruction is associated with an increased risk of left ventricular hypertrophy and late hypertension [14] but also lower rates of reintervention on the descending aorta [7]. However, for patients younger than the age of five, the risk of reintervention is decreased for patients who had the initial repair before the age of 1 year [7].

In the literature, phase-contrast magnetic resonance imaging (MRI), also known as velocity-encoded or flow MRI, has been applied as innovative application to analyse aortic flow haemodynamics in healthy controls and patients. For example, within CoA patients, flow MRI is utilised to examine the pulse wave velocity (PWV), a surrogate marker for the aortic wall stiffness, and WSS. These studies [15–19] demonstrated an increased aortic arch PWV in surgically corrected CoA patients compared to healthy controls. For WSS, both increased [20] and decreased [18] time-averaged WSS were observed in CoA patients compared to healthy control. Surprisingly, only one article was found describing the effect of age at curative reconstruction on the PWV and WSS [19]. However, no discrimination was made on the aorta valve morphology and absence of recurrent obstruction between these patients when evaluating the effect of age at curative reconstruction.

Therefore, this study was aimed at evaluating the effect of age at curative reconstruction on the aortic wall stiffness expressed as PWV and the haemodynamic load expressed as peak WSS in adolescent patients with corrected CoA. Also, effects of valve morphology and presence of reobstruction were evaluated. Our hypothesis is that a correction for CoA patients at younger age may result in less aortic wall stiffening and lower peak haemodynamic load, as a result of the shorter period of hypertension in the arterial system upstream of the coarctation site and exposure to abnormal flow patterns distal to the obstruction.

## Methods

### Study population

This prospective study protocol was approved by the Medical Ethics Committee of the Leiden University Medical Center (P14.095), and informed consent was signed by both parents/guardians of all subjects. Children with chromosomal disorder were excluded to preserve homogeneity of the population. Thirty-two patients after surgical CoA repair participated in the study and underwent a cardiovascular magnetic resonance imaging (MRI) examination. These patients were also included in a previous study with the aim to investigate the cardiac autonomic nervous system activity, cardiac function, and their relationship in children after CoA repair [21]. Eventually, eleven of them were excluded due to practical and emotional problems (*e.g*., patient movement and endurance) during the MRI. The included 21 patients were aged 13.7 ± 2.6 years (mean ± standard deviation), including 12 patients with bicuspid aortic valve (BAV) and 9 patients with tricuspid aortic valve (TAV). Mean age at CoA correction was 1.0 ± 1.8 years, performed by end-to-end anastomosis in 16, extended end-to-end anastomosis in 2, and subclavian flap in 1 patient. Only one patient, as result of a recurrent obstruction, underwent a reoperation using an autologous pericardial patch. None of the patients had a clinical indication for reintervention at the time of the MRI examination. The included patients were scanned between September 2015 and May 2016, and time between reconstruction and MRI was 12.6 ± 3.0 years.

The presence of an aortic reobstruction was determined based on the maximal flow velocity in the descending aorta, measured by a suprasternal transthoracic Doppler echocardiogram (VIVID 9, GE Healthcare, Norway) by a single observer (IN), supervised by an experienced clinician (AH) in all patients. The acquired images were stored and analysed offline using the EchoPAC software version 113 (General Electric Healthcare, Horten, Norway). Based on this analysis, the presence of a reobstruction was determined, defined as a maximal flow velocity larger than 2.5 m/s [22]. Using this criterion, the patient group was divided into two groups: twelve without and nine with recurrent obstruction.

### MRI acquisition

The image acquisition consisted of two MRI through-plane phase-contrast MRI sequences to determine aortic PWV and one four-dimensional (4D) flow MRI sequence. MRI for all patients were performed on a 3-T scanner (Ingenia, Philips Healthcare, Best, The Netherlands) using a combination of both a FlexCoverage posterior coil in the table and a dStream Torso anterior coil, together providing up to 32-coil elements for signal reception. Concomitant gradient correction and local phase correction were performed from standard available scanner software.

The PWV was determined from high-temporal through-plane phase-contrast MRI using free breathing with retrospective electrocardiographic gating, for both the proximal aorta (ascending aorta plus aortic arch) and the descending aorta. This was accomplished by measuring the flow velocity through two planes positioned perpendicular to aortic centreline: the first plane intersecting both the ascending and thoracic descending aorta and the second plane intersecting the abdominal descending aorta, defined as proximal PWV and diaphragmatic PWV, respectively. The proximal PWV MRI sequence parameters were as follows: velocity encoding of 200–300 cm/s in feet-head direction, acquired temporal resolution 8.4 ms, reconstructed temporal resolution 4.1 ms (171 ± 24 phases), echo time 2.3 ms, repetition time 4.2 ms, flip angle 20°, field of view 350 × 350 × 8 mm, and acquired spatial resolution 2.8 × 2.8 × 8.0 mm. Acquisition time and heart rate were on average 75 ± 10 s and 83 ± 12 beats per min, respectively. Diaphragmatic PWV MRI sequence parameters were as follows: velocity encoding of 150–250 cm/s in feet-head direction, acquired temporal resolution 8.6 ms, reconstructed temporal resolution 4.1 ms (167 ± 23 phases), echo time 2.4 ms, repetition time 4.3 ms, flip angle 20°, field of view 350 × 350 × 8 mm, and acquired spatial resolution 2.8 × 2.8 × 8.0 mm. Acquisition time and heart rate were on average 78 ± 12 s and 84 ± 11 beats per min, respectively.

The aortic 4D flow MRI sequence used a hemidiaphragm respiratory navigator, a retrospective ECG gating, and a standard non-symmetrical four-point velocity encoding. Sequence parameters were as follows: velocity encoding of 200–350 cm/s in four directions, acquired temporal resolution 34.4 ms, reconstructed temporal resolution 29.2 ms (26 ± 4 phases), echo time 2.4 ms, repetition time 4.3 ms, flip angle 10°, field of view 350 × 350 × 52.5–72.5 mm, acquired spatial resolution 2.5 × 2.5 × 2.5 mm, segmentation factor 2, and sensitivity encoding factor 2 in anterior-posterior direction. Acquisition time was on average 4.9 ± 0.7 min excluding the respiratory compensation. Due to the acceptance window of the respiratory navigator, the actual acquisition time in the scanner approximately doubled.

### Image analysis

**Fig. 1:**
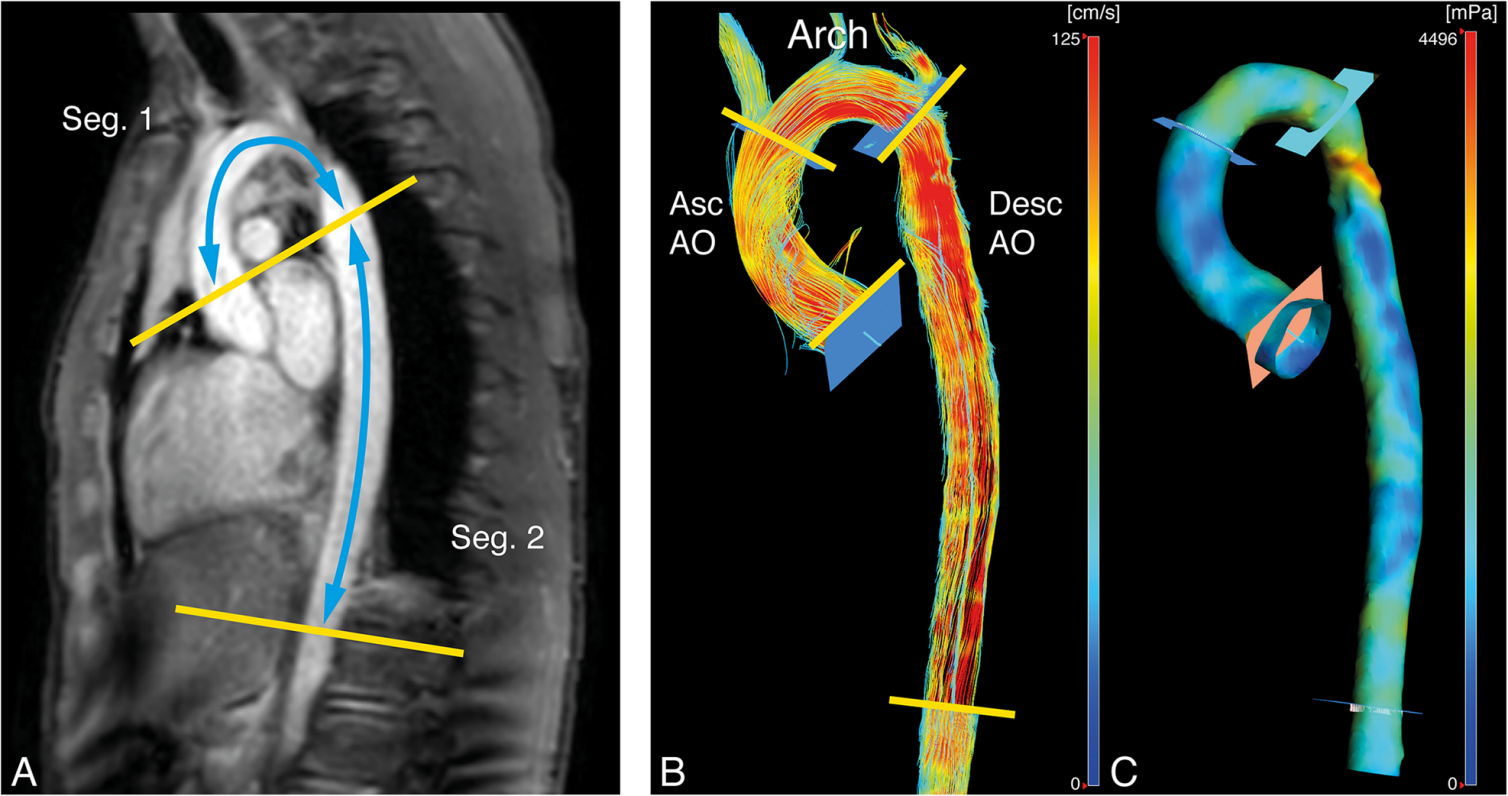
Example of a patient aortic coarctation and tricuspid aortic valve without reobstruction. **a** Pulse wave velocity segments (Seg.): 1, proximal aorta; 2, descending aorta. **b** Wall shear stress segments: *Asc AO*, ascending aorta; *Arch*, aortic arch; *Desc AO*, descending aorta. **c** Three-dimensional magnitude wall shear stress map

The image analysis consisted of two parts to determine aortic PWV and the WSS. In order to obtain the PWV, the acquired proximal PWV and diaphragmatic PWV images firstly were analysed using the in-house developed software MASS (LUMC). This software was used to perform velocity mapping and to measure the length of both aortic segments on a multislice survey of the aorta. Lastly, these quantifications were imported into an in-house developed MATLAB-based application to determine the PWV of the proximal and descending aorta, using the foot-to-foot method (Fig. 1). In all subjects, the PWV image analysis was performed by a single observer (IN) with over 3-year experience in cardiovascular MRI, supervised by an experienced researcher (JW) with over 20 years’ experience in cardiovascular MRI. Additionally, the PWV ratio was also derived from these values, defined as the descending aorta PWV divided by the proximal aortic PWV. This PWV quantification method was previously validated and described in more detail by Grotenhuis et al. [23].

From 4D flow MRI, the WSS was determined using CAAS MR Solutions v5.0 (Pie Medical Imaging, Maastricht, The Netherlands), assuming a constant blood viscosity of 4 mPa s. This software was used to compute the WSS over five time phases and three consecutive aortic segments (Fig. 1): the aortic root plus the ascending aorta, the aortic arch, and the descending aorta (respectively; from the aortic valve to the brachiocephalic artery, from the brachiocephalic artery up and including the left subclavian artery, and from the subclavian artery to the abdominal descending aorta at the level of measurement of the diaphragmatic PWV). This was accomplished by firstly segmenting the aorta on a combined weighted magnitude and velocity image for all five available time phases, incorporating only the aorta and excluding the main branches (*e.g*., the subclavian and carotid arteries). Secondly, the anatomical segmentation planes were manually placed and imported perpendicular to the aortic wall. From proximally to distally on the aorta, these planes were positioned at the aortic valve, proximally against the brachiocephalic artery, distally against the subclavian artery, and 10 cm caudal below the diaphragm. Thirdly, for the five available time phases and each anatomical segment, the maximal WSS was exported from CAAS. Lastly, these maxima over the five time phases were used to determine the peak WSS for each anatomical segment over all five time phases. The WSS image analysis was performed by a single observer (IN) in all patients. The applied method to determine the WSS in the five systolic time phases was previously described and validated on the reproducibility by van der Palen et al. [24].

### Statistical analysis

The statistical analysis was performed using the SPSS v23 software (IBM, Chicago, IL, USA). Differences between groups were compared using the independent sample *t* test or Mann-Whitney *U* test, respectively used for parametric scale data or non-parametric scale and ordinal data. Correlations between variables within groups were evaluated using the Pearson (*r*_P_) and Spearman rank (*r*_S_) correlations, respectively used for parametric scale data or non-parametric scale and ordinal data. The Levene test was used to verify the equality of variance and Shapiro-Wilk test to verify the normality of the data. The absolute correlation coefficient (*r*_P_ or *r*_s_) was classified as follows: 0.30 < |*r*| < 0.50, weak; 0.50 < |*r*| < 0.70, moderate; 0.70 < |*r*| < 0.85, good; and |*r*| > 0.85, strong. All statistical tests were two-tailed, and a *p* value of less than 0.05 was considered significant. Data will be presented as mean values with standard deviations.

## Results

**Fig. 2:**
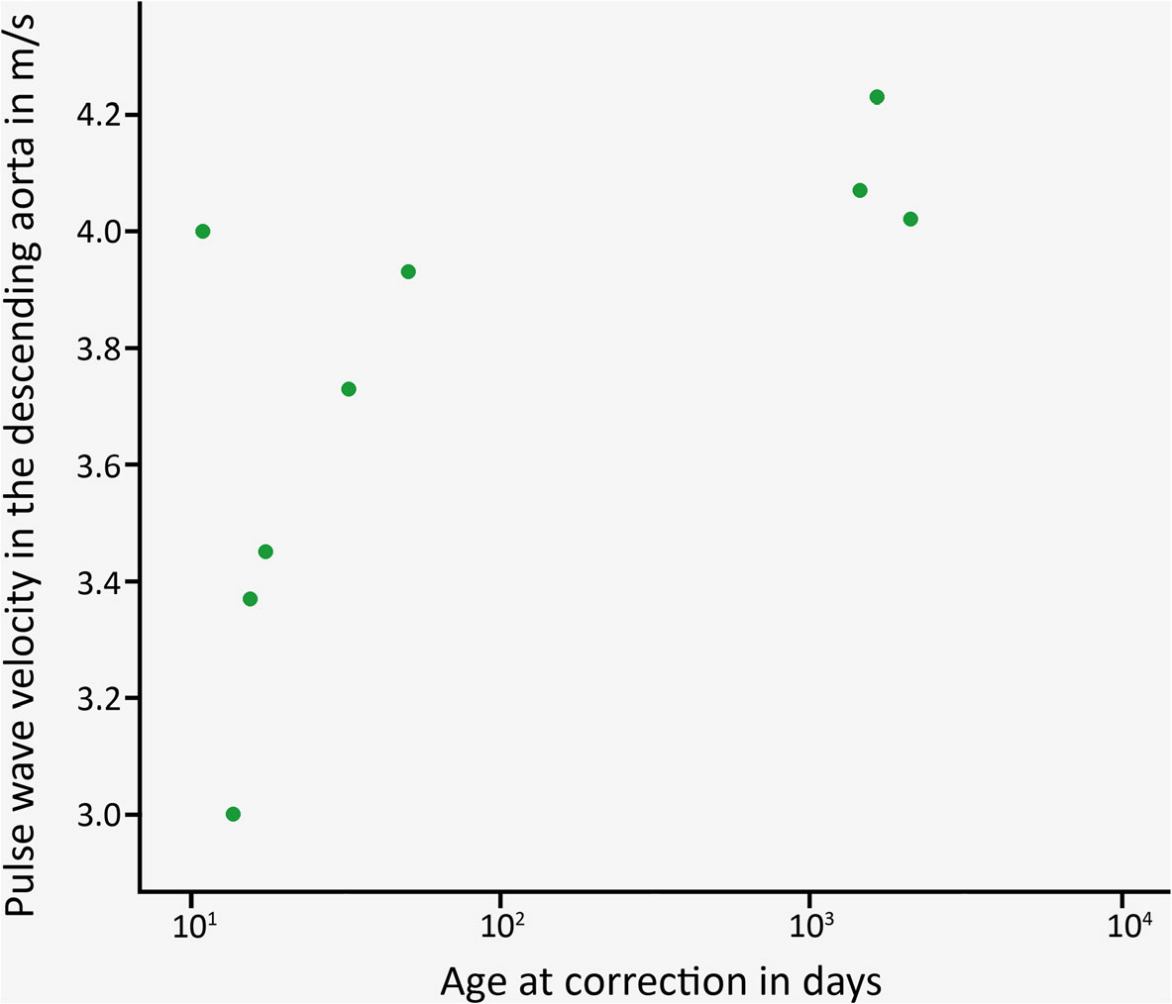
Plot of the descending aorta pulse wave velocity over the age at correction for the tricuspid aortic valve patient subgroup

**Fig. 3:**
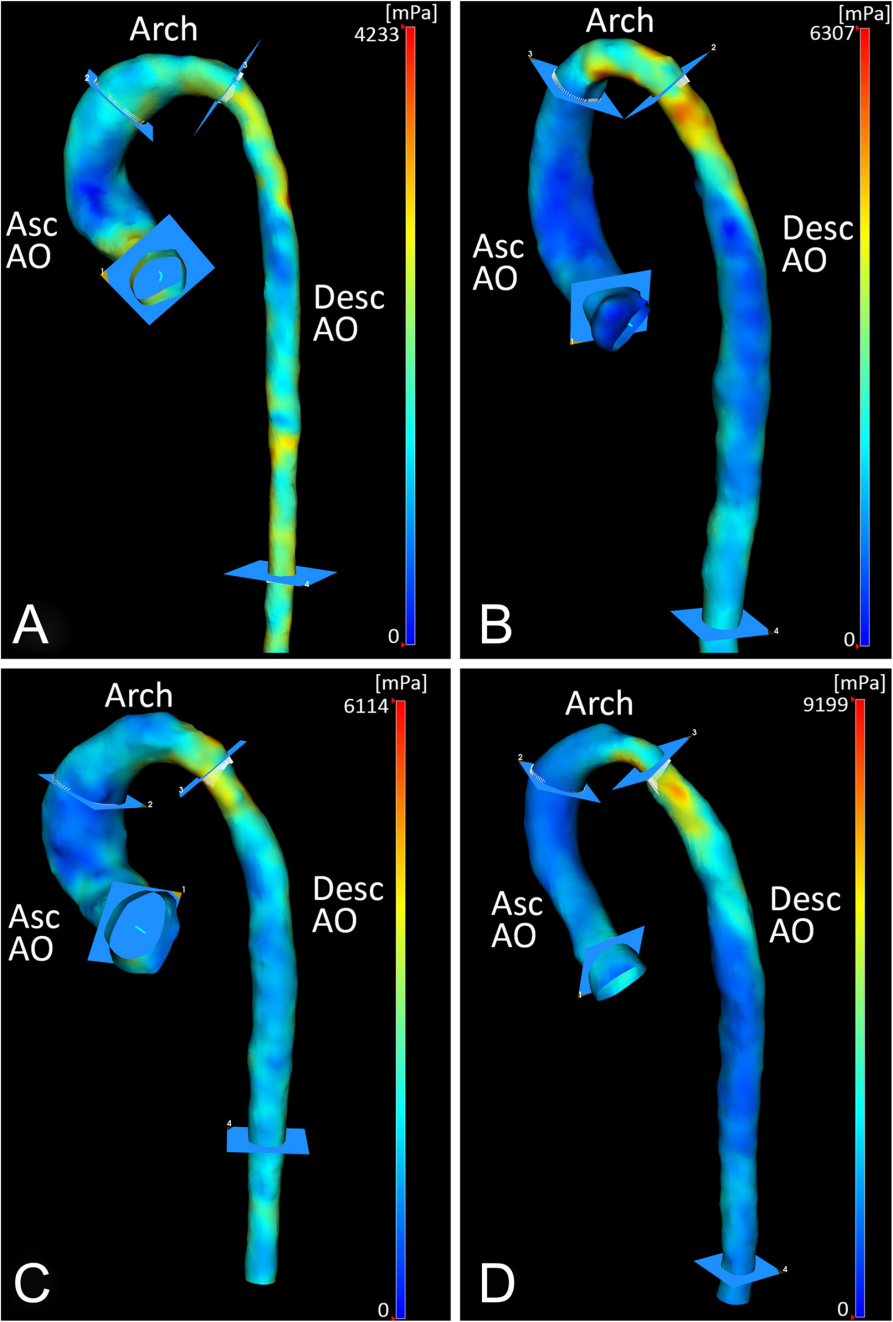
Three-dimensional magnitude wall shear stress maps of patients with aortic coarctation subdivided on both the aortic valve morphology and the presence of reobstruction, incorporating the anatomical segments: *Asc AO*, ascending aorta; *Arch*, aortic arch; *Desc AO*, descending aorta. **a** Example of patient with tricuspid aortic valve without reobstruction. **b** Example of patient with tricuspid aortic valve with reobstruction. **c** Example of patient with bicuspid aortic valve without reobstruction. **d** Example of patient with bicuspid aortic valve with reobstruction

Characteristics of the CoA patients and subgroups are shown in Table 1. Non-significant correlations within the entire patient population (*n* = 21) were found between the following parameters: the age at correction and MRI, PWV in the proximal and descending aorta, PWV ratio, and peak WSS in the ascending aorta, aortic arch, and descending aorta. And, for the same parameters, non-significant group differences between the BAV and TAV subgroups were found (respectively, *n* = 12 and *n* = 9). For patients with a TAV, a significant good correlation was found between age at correction and descending aorta PWV (*r*_**s**_ = 0.80, *p* = 0.010, Fig. 2), indicating higher values of descending aorta PWV for patients with a TAV that underwent correction of CoA at an older age. Such a correlation was absent for patients with BAV. Between the subgroups without reobstruction and with reobstruction (respectively, *n* = 12 and *n* = 9), significant differences were found for the peak WSS in the aortic arch (3.9 ± 1.3 Pa *versus* 6.5 ± 2.2 Pa, respectively; *p* = 0.003) and descending aorta (5.0 ± 1.3 Pa *versus* 6.7 ± 1.1 Pa, respectively; *p* = 0.005), indicating higher peak WSS values for patients with a reobstruction proximally and distally to the lesion. The statistical analysis within subgroups subdivided on both the aortic valve morphology and the presence of reobstruction was not performed, due to small population sizes within these subgroups. Examples of three-dimensional magnitude WSS maps of patients, subdivided on the aortic valve morphology and the presence of reobstruction, are shown in Fig. 3. The examples demonstrate higher peak WSS in the aortic arch and descending aorta for patients with reobstruction compared to those without reobstruction.

**Table 1:** Patient and subgroup characteristics

	Patients	TAV	BAV	No reobstruction	Reobstruction
Populations size	21	9	12	12	9
Age at reconstruction (years)	1.0 ± 1.8	1.6 ± 2.4	0.5 ± 1.1	0.5 ± 1.2	1.6 ± 2.4
Age at MRI (years)	13.7 ± 2.6	13.1 ± 2.5	14.1 ± 2.7	12.9 ± 2.9	14.7 ± 1.7
Time between reconstruction and MRI (years)	12.6 ± 3.0	11.5 ± 2.9	13.5 ± 2.8	12.3 ± 2.9	13.1 ± 3.3
Body mass index (kg/m^2^)^a^	20.3 ± 4.7	18.7 ± 1.8	21.5 ± 5.8	20.3 ± 5.8	20.3 ± 2.7
Body surface area (m^2^)	1.5 ± 0.3	1.5 ± 0.2	1.6 ± 0.3	1.5 ± 0.3	1.6 ± 0.3
Gender (male/female)	11/10	2/7	9/3	5/7	6/3
PWV proximal aorta (m/s)	4.8 ± 1.4	5.0 ± 1.3	4.7 ± 1.5	4.8 ± 1.7	4.9 ± 0.9
PWV descending aorta (m/s)	3.7 ± 0.8	3.75 ± 0.4	4.1 ± 1.1	3.7 ± 0.5	4.2 ± 1.2
PWV ratio	0.9 ± 0.3	0.8 ± 0.2	0.9 ± 0.3	0.9 ± 0.3	0.9 ± 0.3
Peak WSS ascending aorta (Pa)	5.3 ± 1.4	4.8 ± 1.1	5.7 ± 1.4	4.9 ± 0.9	5.9 ± 1.7
Peak WSS aortic arch (Pa)	5.0 ± 2.1	4.6 ± 2.6	5.3 ± 1.8	3.9 ± 1.3	6.5 ± 2.3
Peak WSS descending aorta (Pa)	5.7 ± 1.4	5.3 ± 1.3	6.0 ± 1.5	5.0 ± 1.3	6.7 ± 1.1

Data presented as mean ± standard deviation. *BAV* Bicuspid aortic valve, *PWV* Pulse wave velocity, *TAV* Tricuspid aortic valve, *WSS* Wall shear stress

^a^According to Dubois formula

## Discussion

In this study, the effect of age at curative reconstruction on the aortic wall stiffness expressed as PWV and the haemodynamic load by viscous friction on the arterial wall, expressed in peak WSS, was evaluated in adolescent patients with corrected CoA. Also, the effects of the aortic valve morphology and presence of reobstruction were evaluated. The main findings of this study were as follows: (1) a significant positive correlation between age at correction and PWV in the descending aorta for the TAV subgroup and (2) a significant difference in peak WSS in the aortic arch and descending aorta between the subgroup without reobstruction and the subgroup with reobstruction.

The observed positive correlation for TAV subgroup between the age at correction and descending aorta PWV suggests that a prolonged period of abnormal haemodynamic exposure may result in increased aortic wall stiffening. Specifically for TAV patients, this is in line with the hypothesis that for CoA patients, a curative reconstruction at a younger age will result in less wall stiffening, thus a lower PWV. This result should be interpreted with caution, since no correlation was found within the entire patient group nor in the BAV subgroup. The absence of a comparable correlation for these groups may be explained by the abnormal haemodynamics of BAV patients [25], potentially resulting in different disease progressions for both aortic valve morphologies. Therefore, the significant finding in the TAV subgroup (notably, smaller in size than BAV subgroup) represents an evaluation of the CoA correction for patients selected on valve morphology. The abnormal haemodynamics of BAV patients results in more diversity of flow patterns within the entire patient population and subsequently may decrease the probability of detecting a significant effect. However, Voges et al. [19] observed a weak but significant positive correlation (*r* = 0.33) between the age at correction and the descending aorta PWV within their entire patient group, incorporating 16 BAV and 35 TAV patients. This effect presumably may be influenced by substantial larger group of TAV patients compared to BAV patients. Unfortunately, these authors did not statistically analyse this correlation separately within both aortic valve morphology subgroups.

The observed peak WSS differences in the aortic arch and descending aorta between the patients without and with reobstruction imply that local luminal narrowing results in an increased haemodynamic load on the aortic wall proximally and distally to the lesion. This effect was predominant within the aortic arch. Multiple studies indicated that endothelium lining properties are highly sensitive to the applied WSS on the vessel wall, which promotes adaptive dilation or structural remodelling of the artery wall during high WSS [11]. Therefore, different disease progression could potentially be expected for patient with and without postoperative obstructions. This endorses the initial curative reconstruction and longitudinal follow-up examination of corrected CoA patients. However, the surgical reconstruction is associated with the formation of abnormal postoperative aortic haemodynamic due to aorta compliance [8] and geometry [26, 27] modifications. For example, it has been demonstrated that the postoperative aortic arch geometry in CoA patients affects the PWV [27] and peak WSS magnitude and location [26]. Additionally, the presence of an aortic reobstruction was defined as a maximal flow velocity larger than 2.5 m/s. This criterion is arbitrary since peak flow velocity alone does not always discriminate patients without obstruction and those with obstruction [22]. Therefore, a single Doppler echocardiogram measurement will probably not be the decisive factor for reintervention in most clinical centres.

The present study has several limitations. Firstly, the number of CoA patients was limited by the available data, resulting in a relatively small population size and statistical power which also made the comparison of subgroups based on multiple patient characteristics difficult. Still, we were able to detect statistical significant findings for these limited group sizes. Secondly, the study only incorporated patients and no healthy controls, excluding the possibility to compare our results with reference values. Thirdly, the single-centre design limited the patient diversity, resulting in a relatively small variation of age at CoA correction. Fourthly, the severity of CoA prior to reconstruction is an important confounder. However, information of the severity of CoA was not available and therefore not involved in the statistical evaluation.

In conclusion, the association between the age at correction and descending aorta PWV for TAV patients suggests that a prolonged period of abnormal haemodynamic exposure may result in increased aortic wall stiffening. The increased peak WSS as results of a reobstruction possibly promotes different disease progression, which endorse longitudinal follow-up examination of corrected CoA patients.

## Abbreviations

4D: Four-dimensional
BAV: Bicuspid aortic valve
CoA: Aortic coarctation
MRI: Magnetic resonance imaging
PWV: Pulse wave velocity
TAV: Tricuspid aortic valve
WSS: Wall shear stress
